# The complete mitogenome of the inarticulate brachiopod *Glottidia pyramidata* reveals insights into gene order variation, deviant ATP8 and mtORFans in the Brachiopoda

**DOI:** 10.1080/23802359.2021.1966342

**Published:** 2021-08-18

**Authors:** Thierry Niaison, Davide Guerra, Sophie Breton

**Affiliations:** Department of Biological Sciences, University of Montreal, Montreal, Canada

**Keywords:** Brachiopoda, comparative mitogenomics, gene order ATP8, mtORFans

## Abstract

Brachiopods are a clade of marine organisms with a tremendously diverse and abundant fossil record but with fewer than 500 species extant today. Even if a better understanding of their biology and genetics could help to test hypotheses about their impressive decline, knowledge of genetics and evolutionary genomics in extant brachiopods is very poor. Here, we present the complete mitochondrial genome sequence of the inarticulate *Glottidia pyramidata*, an eastern North American extant representative of the phylum Brachiopoda. Besides the general characteristics of the sequenced mitogenome, we present its unusual features such as deviant ATP8 protein sequence and supernumerary ORFs, and also unique gene order, considering the available genome sequences of other brachiopod species.

Also known as lamp shells or the ‘other’ bivalves, brachiopods are a phylum of marine invertebrates with a rich paleontological history, with more than 30,000 extinct species known to science (Thayer 1985; Carlson 2016). Even though they resemble bivalved mollusks, brachiopods are in fact bivalved lophophorates. Unlike their diverse and abundant ancestral counterparts, fewer than 500 species are alive today and they live largely hidden, at great depths and locations with low diversity and abundance (Carlson 2016). Competition from ‘mussel-like bivalves’ has been proposed as a plausible cause of the spectacular decline of this animal group in the end-Permian mass extinction (Thayer 1985). A better understanding of the biology and genetics of the few extant species may help to test hypotheses about this impressive decline, but knowledge of genetics and evolutionary genomics in extant brachiopods is still relatively poor (Carlson 2016), including at the mitochondrial level.

Interestingly, some of the few brachiopod mitochondrial DNAs (mtDNAs) sequenced to date resemble those of bivalves with DUI (i.e. with doubly uniparental inheritance of mtDNA characterized by the presence of two sex-associated mtDNA lineages inherited through males and females, respectively; Breton et al. 2007; Stewart et al. 2020). As for DUI bivalves (e.g. Breton et al. 2010, 2011a, 2011b, 2014; Milani et al. 2013; Lubośny et al. 2018; Guerra et al. 2019; Capt et al. 2020), brachiopods present important variation in mtDNA size (17 to >28 kb) and gene arrangement, with longer and deviant protein-coding sequences compared to their homologues in other animals (e.g. longer *cox2* gene and deviant ATP8 protein sequences) as well as supernumerary open reading frames (ORFs) or mtORFans (ORFs without recognizable homologies to other known genes) (Helfenbein et al. 2001; Endo et al. 2005; Luo et al. 2015; Karagozlu et al. 2017a). There are seven complete brachiopod mitogenomes available to date, three of them are from *Lingula anatina* (order Lingulida), and the four others are from species from the order Terebratulida, which contains >75% of all extant brachiopod species (Emig et al. 2013). Three other orders exist in this phylum: Craniida, Rhynchonellida, Thecideida (Emig et al. 2013). In the traditional two-class system, Terebratulida + Rhynchonellida + Thecideida constitute the class Articulata (brachiopods with two valves connected by a tooth and socket hinge) and Lingulida + Craniida the class Inarticulata (unhinged valves connected by muscles alone) (Carlson 2016). It is mainly the inarticulate *L. anatina* that presents unusual mitochondrial features, such as intraspecific variation in size and arrangement, and mtORFans (Endo et al. 2005; Luo et al. 2015; Karagozlu et al. 2017a). This raises the question if some of these features are also shared by other inarticulate species, thus highlighting the need for more complete mtDNAs to expand our knowledge on brachiopod mitogenomics.

Here, we present the complete mitogenome of the inarticulate brachiopod *Glottidia pyramidata* Stimpson 1860 (Brachiopoda: Inarticulata). Four adult specimens were collected by Gulf Specimen Marine Laboratories and Aquarium in June 2016 in the Dickerson Bay (USA; near N30.023633 W84.385280) and sent alive to the Université de Montréal. Each individual was dissected and sexed by inspecting the gonads under a light microscope (×100) for the presence of eggs or sperm, three females and one male were unambiguously sexed and total DNA was extracted from one male gonad, sequenced, assembled, and annotated following Guerra et al. (2018). The specimen and DNA sample were deposited in the Department of Biological Sciences Tissue & DNA Collection at the Université de Montréal (https://bio.umontreal.ca/english/home/; specimen code B1408 and DNA sample code B1408g; person in charge Breton S, s.breton@umontreal.ca). Since brachiopods present characteristics that resemble those of DUI bivalves, we first looked for the presence of DUI in *G. pyramidata*, but our results revealed only one set of mitochondrial contigs (consistent with the existence of only one mitochondrial lineage), suggesting that it might possess a strictly maternal mitochondrial transmission like other metazoans (Breton and Stewart 2015). The mitogenome sequence has been deposited in GenBank (accession number MW732171).

**Figure 1.**
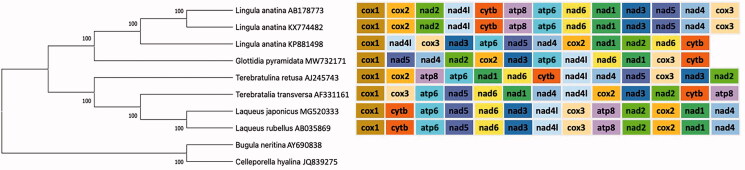
Maximum-likelihood phylogeny of the inarticulate *Glottidia pyramidata* in the phylum Brachiopoda. The tree was obtained with MEGAX and is based on concatenated sequences of 12 protein-coding genes (*atp8* was excluded) from eight brachiopod species and two outgroups chosen from the phylum Bryozoa. GenBank accession numbers are presented after species names. The number below or above the branches indicate bootstrap support values above 95%. All known gene orders of Brachiopoda were mapped on the obtained phylogeny.

The complete mtDNA of *G. pyramidata* is 19,709 bp long, which is larger than the mtDNAs of the articulate brachiopods *Terebratulina retusa* (15,451 bp; Stechmann and Schlegel 1999), *Terebratalia transversa* (14,291 bp; Helfenbein et al. 2001), *Laqueus rubellus* (14,017 bp; Noguchi et al. 2000), and *Laqueus japonicus* (14,267 bp; Karagozlu et al. 2017b), but in the range of the sizes observed for the inarticulate *L. anatina* (17,970 bp, Luo et al. 2015; 25,790 bp, Karagozlu et al. 2017a, and 28,818 bp, Endo et al. 2005). It contains 36 genes, which are all located on the same strand as in other brachiopod species. The annotation of *atp8* is unsure, otherwise all other typical mitochondrial genes, i.e. 12 coding for proteins, 22 for tRNAs, and two for rRNAs, were retrieved and annotated without any ambiguities. The *atp8* gene has potentially been localized in an unassigned region preceding *atp6* (as for the inarticulate *L. anatina*), and it is shorter (43aa) than what has been found in other brachiopods (51aa to 59aa). It does not possess the typical MPQL amino acid signature present at the N-terminus of metazoan ATP8 (only *L. anatina* also presents this feature), but it is characterized by a hydrophobic N-terminus domain like typical ATP8 proteins (Breton et al. 2010). Moreover, as *L. anatina*, the mitogenome of *G. pyramidata* contains mtORFans, which are found between the genes *nad4L* and *nad6* (i.e. three mtORFans of 200, 99, and 64 amino acids).

For our phylogenetic analysis, all brachiopod mitogenomes publicly available, as well as two outgroup mitogenomes (the bryozoans *Bugula neritina* [AY690838] and *Celleporella hyalina* [JQ839275]) were retrieved from GenBank (01-07-2020) and their 12 protein-coding genes (*atp8* was excluded) were translated with the invertebrate mitochondrial genetic code and individually aligned using MEGAX (Kumar et al. 2018) and the MUSCLE algorithm (Edgar 2004). These alignments were afterwards concatenated with MEGAX and used to construct a maximum-likelihood (ML) tree using MEGAX and the JTT model for amino acid substitution. We mapped all known brachiopod gene orders on the phylogeny. Our results show that the inarticulate *G. pyramidata* clustered together with the inarticulate *L. anatina* with high support value, and that the articulate brachiopods cluster together (Figure 1). The gene order in *G. pyramidata* is unique among brachiopod species sequenced to date, adding to the gene order variability previously reported in this group (Luo et al. 2015).

## Data Availability

The genome sequence data that support the findings of this study are openly available in GenBank of NCBI at https://www.ncbi.nlm.nih.gov under the accession no. MW732171. The associated BioProject, SRA, and Bio-Sample numbers are PRJNA737150, SRP323932, and SAMN19679991, respectively.
